# Combined Amoebicidal Effect of Atorvastatin and Commercial Eye Drops against *Acanthamoeba castellanii* Neff: In Vitro Assay Based on Mixture Design

**DOI:** 10.3390/pathogens9030219

**Published:** 2020-03-17

**Authors:** Ines Sifaoui, Eulalia Capote Yanes, María Reyes-Batlle, Rubén L. Rodríguez-Expósito, José E. Piñero, Jacob Lorenzo-Morales

**Affiliations:** 1 Instituto Universitario De Enfermedades Tropicales y Salud Pública de Canarias, Universidad de La Laguna, Avda. Astrofísico Fco. Sánchez, S/N, La Laguna, Tenerife, 38203 Islas Canarias, Spain; laliacapote@hotmail.es (E.C.Y.); mreyesba@ull.edu.es (M.R.-B.); ruben_rguez_exposito92@hotmail.com (R.L.R.-E.); jmlorenz@ull.edu.es (J.L.-M.); 2 Departamento de Obstetricia, Ginecología, Pediatría, Medicina Preventiva y Salud Pública, Toxicología, Medicina Legal y Forense y Parasitología, Universidad De La Laguna, La Laguna, Tenerife, 38203 Islas Canarias, Spain; 3 Servicio de Oftalmología, Hospital Universitario Nuestra Señora de la Candelaria, Canarias, Tenerife, 38010 Islas Canarias,, Spain

**Keywords:** *Acanthamoeba* keratitis, chemotherapy, atorvastatin, Diclofenaco-lepori, mixture design

## Abstract

The establishment of an effective therapeutic agent against *Acanthamoeba* keratitis (AK), remains until present, an issue to be solved due to the existence of a cyst stage in the life cycle of *Acanthamoeba*. Moreover, the effectiveness of the current standard therapeutic agents varies depending on the tested *Acanthamoeba* strains and its resistance pattern. In the present study, two 10-point augmented simplex-centroid designs were used to formulate a three-component mixture system using water, atorvastatin, and Diclofenaco-lepori or Optiben. The amoebicidal effects and in vitro-induced toxicity in a eukaryotic cell line were determined for all experiments. The optimal mixture to inhibit the parasite without inducing toxicity was established in the first plan as 30% Optiben, 63.5% atorvastatin, and 3.1% water. As for the second experimental design, the optimal mixture to inhibit *Acanthamoeba* with lower toxicity effect was composed of 17.6% Diclofenaco-lepori and 82.4% atorvastatin.

## 1. Introduction

*Acanthamoeba*, a member of the free-living amoeba group, is a ubiquitous protist and opportunistic parasite that has been isolated from different habitats, such as soil, water, air, dust, drinking water, sea water, and recreational water (home aquaria, swimming pools) [1,2,3]. Current molecular classification is based on the sequences of the nuclear small ribosomal subunit gene (18S rDNA) that classifies *Acanthamoeba* strains within 22 genotypes known as T1 to T22 [4]. Although genotype T4 is the most common in both clinical and environmental samples, other genotypes—namely T2, T3, T5, T6, T11, T13, and T15, among others—have been also related to AK and amoebic encephalitis [5,6].

Currently, the treatment of *Acanthamoeba* infections consists of a combination of biguanides, amidines, and azoles. Moreover, this therapeutic regimen has limited efficacy due to the toxicity of the drugs, the requirement for long-term treatment, variable efficacy between strains or species, and the presence of a resistant cyst form. Recent advances reported by our group have highlighted the potent amoebicidal activity of atorvastatin without any cytotoxicity issues [7,8]. Atorvastatin belongs to the family of statins and it is a synthetic HMG-CoA (3-hidroxi-3-metilglutaril-coenzima A) reductase inhibitor that causes a reduction of cholesterol levels.

**Figure 1:**
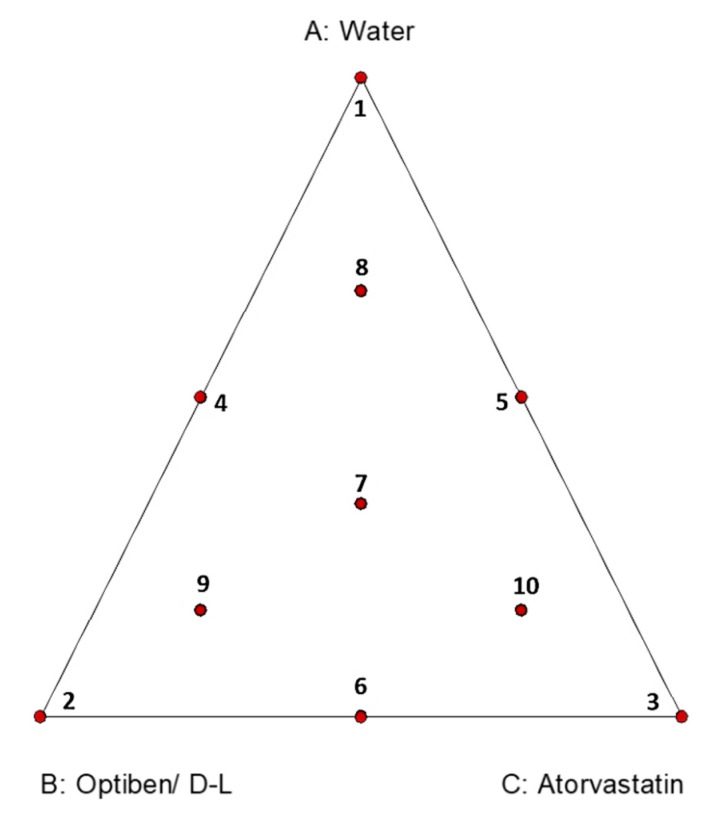
Ternary plot of a three-compound combination containing the 10 points or experiments to define a surface.

Recently, the experimental design approach has been widely used in order to determine the relationship between several factors and its effect on a system. The main advantage to use such a strategy is to carry out a project and reach the objective while minimizing the project’s cost and the number of experiments required [9]. Experimental design, and in particular the mixture designs, was therefore used to determine the occurrence of interactions between some pharmaceutical excipients and to find the optimal constituent proportions for the final formulation [10]. Mixture experimental designs were first reported by Scheffé in 1958 [11,12]. This approach is widely used in industrial product formulation (chemical, textile fibers), food industries, and in the production of pharmaceutical drugs. In fact, this design is used to find a factor combination that allows an optimal response profile to be reached. In the case of screenings, in general, the experimental data fit a linear model and in the case of optimization, the design data usually fit a quadratic or special cubic model [13]. This mixture plan is constituted by formulations of three pure mixtures (one for each ingredient): three binary blends (one for each possible two-ingredient blend); three complete blends (all ingredients included but not in equal proportions); and one center point (all components included in equal proportions), as shown in Figure 1. Moreover, the sum of all the mixture components in each formulation is always equal to 1 [14].

The aim of the present work was to apply the mixture design to evaluate the effect of combining atorvastatin (stock solution at 20 µM) with two commercial eye drops: Optiben (CINFA, Pomplona, Spain) and Diclofenaco-lepori (D-L) (1 mL/mL) (Angelini Farmacéutica, Barcelona, Spain) against *A. castellanii* Neff and a murine macrophages cell line J774-A1 [15]. Moreover, the objective was to study the interaction between the different mixture components using the minimum combination and experiments.

## 2. Results and Discussion

### 2.1. Model Fitting, Regression Analysis

#### 2.1.1. Mixture Design of Atorvastatin, Optiben, and Water

In this study, a drug mixture to inhibit the growth of *Acanthamoeba castellanii* Neff, without inducing toxicity effects, was investigated. For this purpose, a 10-point augmented simplex-centroid design was used to formulate three-component mixture systems comprised of atorvastatin, Optiben, and water (Table 1). The fluorescence emitted by *Acanthamoeba castellanii* Neff and murine macrophages was selected as the responses for the present design.

**Figure 2:**
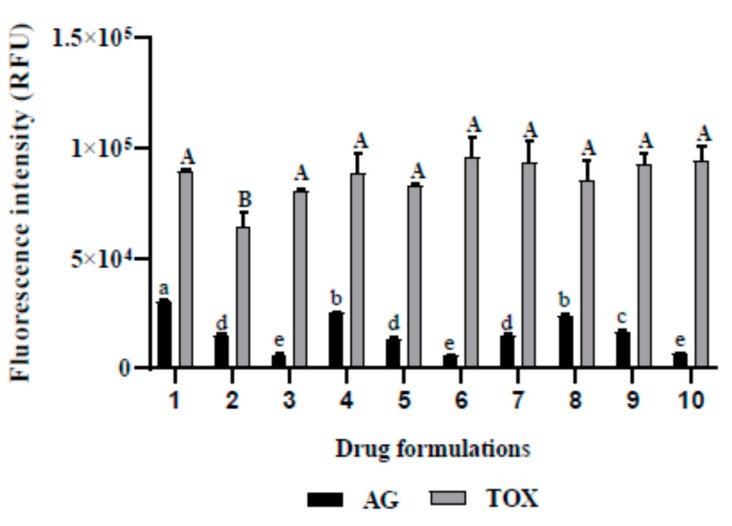
In vitro effect of the different drug formulations when incubated with *A. castellanii* Neff (AG) and the murine macrophages cell lines (TOX). (**1**) water; (**2**) Optiben; (**3**) atorvastatin; (**4**) water (1/2) + Optiben (1/2); (**5**) water (1/2) + atorvastatin (1/2); (**6**) Optiben (1/2) + atorvastatin (1/2); (**7**) water (1/3) + Optiben (1/3) + atorvastatin (1/3); (**8**) water (2/3) + Optiben (1/6) + atorvastatin (1/6); (**9**) water (1/6) + Optiben (2/3) + atorvastatin (1/6); (**10**) water (1/6) + Optiben (1/6) + atorvastatin (2/3). Data with different lowercase letters (a–e) for AG are significantly different (*p* < 0.05). Data with different uppercase letters (A–B) for toxicity levels are significantly different (*p* < 0.05).

When considering the inhibition of *A. castellanii* Neff growth, the use of different compounds slightly enhanced the drug activity, as shown in Figure 2. Even though the Relative Fluorescence Units (RFU) decreased from 6349 to 5570 for experiment 3 and 6, respectively, this difference was not statistically significant. Regarding the induced toxicity in the tested cell line of murine macrophages, we noticed that excepting the formulation number 2, the variation in the drug mixture did not statistically cause toxicity issues.

The three components were used alone or in combination. Herein, the experimental mixture design was used to achieve the optimum composition of drug solution to eliminate this parasite and to study the drug interaction between the different components. The experimental data were used to calculate the coefficients of the model equation. In this case, a special cubic model was defined to describe both responses. The coefficient of determination (*R*^2^) and *p*-values were used to verify the model fit quality. It was considered that a high regression coefficient and a small *p*-value for any of the terms in the model would indicate a more significant effect on the response variables. Regression coefficients and coefficients of determination were calculated and are provided in Table 2.

Both responses have determination coefficients (*R*^2^) greater than 0.95, demonstrating that the model and the equation could be robustly developed. Among the independent variables, all the linear coefficients presented positive values. However, the interaction between Optiben and atorvastatin (b_23_) was negative. Both drugs together exerted higher amoebicidal effects due to possible synergistic effects.

#### 2.1.2. Mixture Design of Atorvastatin, Diclofenaco-lepori, and Water

Atorvastatin, Diclofenaco-lepori (D-L), and water alone or in combination were used to study their effect on the growth of *A. castellanii* Neff and induced toxicity levels, as shown in Table 3. The fluorescence for amoebicidal and cytotoxicity activities were selected as the responses for the combination of the independent variables.

**Figure 3:**
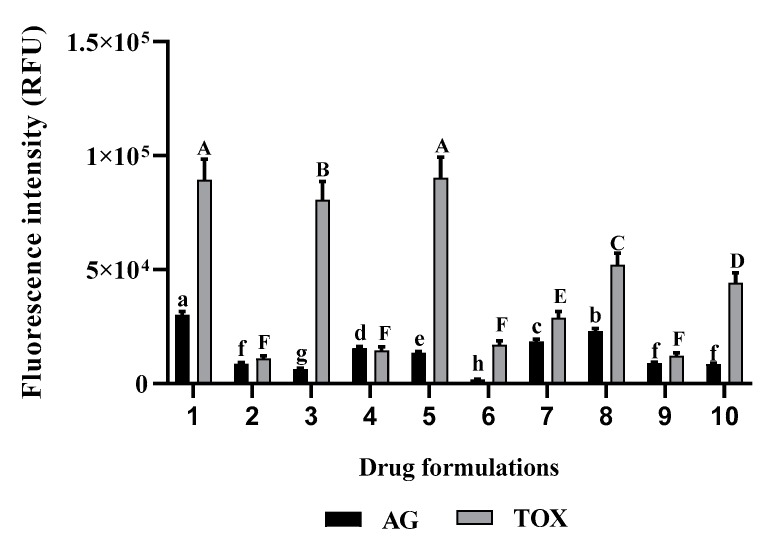
In vitro effect of the different drugs formulations against *A. castellanii* Neff and induced toxicity in the tested murine macrophage cell line. (**1**) water; (**2**) Diclofenaco-lepori (D-L); (**3**) atorvastatin; (**4**) water (1/2) + D-L (1/2); (**5**) water (1/2) + atorvastatin (1/2); (**6**) D-L (1/2) + atorvastatin (1/2); (**7**) water (1/3) + D-L (1/3) + atorvastatin (1/3); (**8**) water (2/3) + D-L (1/6) + atorvastatin (1/6); (**9**) water (1/6) + D-L (2/3) + atorvastatin (1/6); (**10**) water (1/6) + D-L (1/6) + atorvastatin (2/3). Data with different lowercase letters (a–g) for AG are significantly different (*p* < 0.05). Data with different uppercase letters (A–F) for toxicity levels are significantly different (*p* < 0.05).

Considering the mixture of D-L and atorvastatin, the variation in drug formulations significantly affected the growth of *A. castellanii* Neff and murine macrophages (*p* < 0.05). The use of those drugs in a mixture enhance their amoebicidal capacity, however, a rich mix in D-L increases the cytotoxicity of the formulation towards the murine macrophages (Figure 3).

The experimental mixture design was applied to achieve the optimum composition of drug solution to eliminate this parasite and to study the drug interaction between the mix components. A quadratic model was defined for the present design. The coefficients of determination (*R*^2^) and *p*-values were used to verify the model fit quality. Regression coefficients and coefficients of determination were calculated and are provided in Table 4.

Both responses have an *R*^2^ higher than 0.95, demonstrating that the model and equation could be developed with confidence. Among the independent variables, the interaction between both drugs had a negative effect on both responses, due to the toxicity of D-L. In fact, as can be observed in Table 3, macrophages treated only with Diclofenaco-lepori emitted the lowest fluorescence due to cellular death—the fluorescence of AlamarBlue is proportional to the cellular growth rate. This assay is based on the reduction of the blue non-fluorescent dye resazurin to the pink-colored, highly fluorescent resorufin [16]. Furthermore, Ayaki et al. (2008) reported that eye drops based on diclofenac were toxic to corneal epithelium [17]. As for the *Acanthamoeba* growth, we report the existence of synergy between the D-L and atorvastatin.

### 2.2. Ternary Plots Analysis

**Figure 4:**
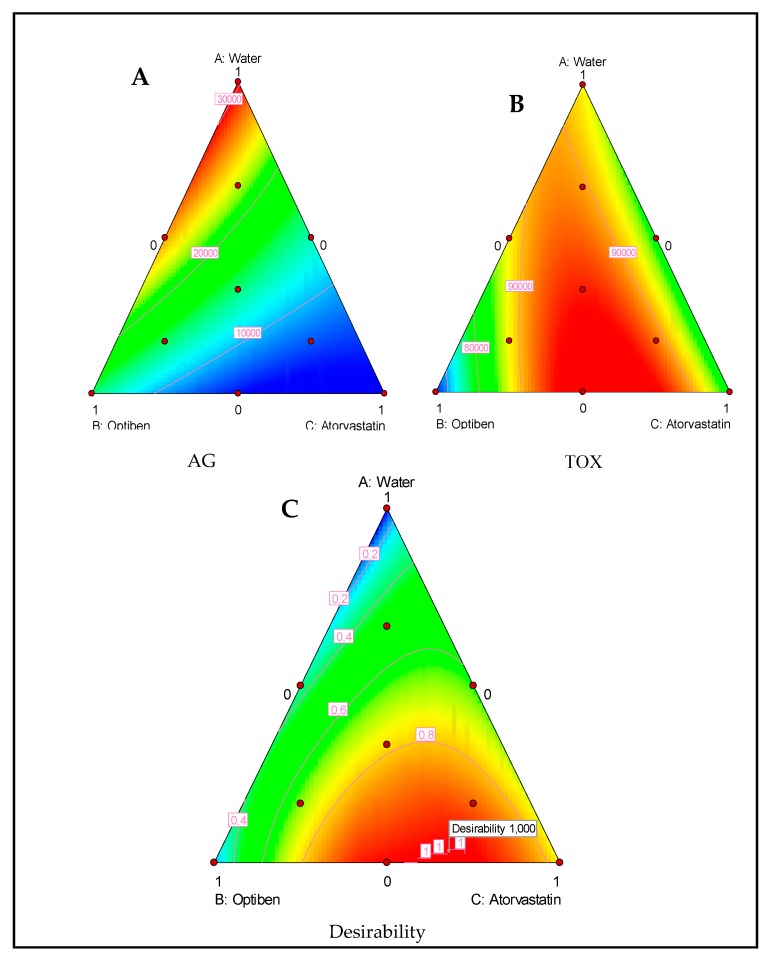
Ternary plots of mixture between water, Optiben, and Atorvastatin showing the effect of those components on *Acanthamoeba* growth (AG) (**A**) and induced toxicity (TOX) (**B**). Ternary plot for the desirability function showing the optimal drug mixture to inhibit the parasite without inducing toxic effects (**C**).

Figure 4A,B represents the effect of the drug composition on the anti-*Acanthamoeba* action and the induced toxicity. The drugs presented high activity against the parasite but low toxicity against the murine macrophage cell line. Additionally, the lowest fluorescence was produced by atorvastatin when compared to Optiben. Moreover, the inhibition of *Acanthamoeba* interpreted by the decrease in the fluorescence was proportional to the quantity of atorvastatin. Regarding cytotoxicity levels, even though all the linear effects were non-significant against the cell line growth rate, a mix of the three components or a mix of the eye drop Optiben and atorvastatin were slightly less toxic than the pure components. Moreover, the correspondent’s coefficient for this interaction was statistically significant and positive.

**Figure 5:**
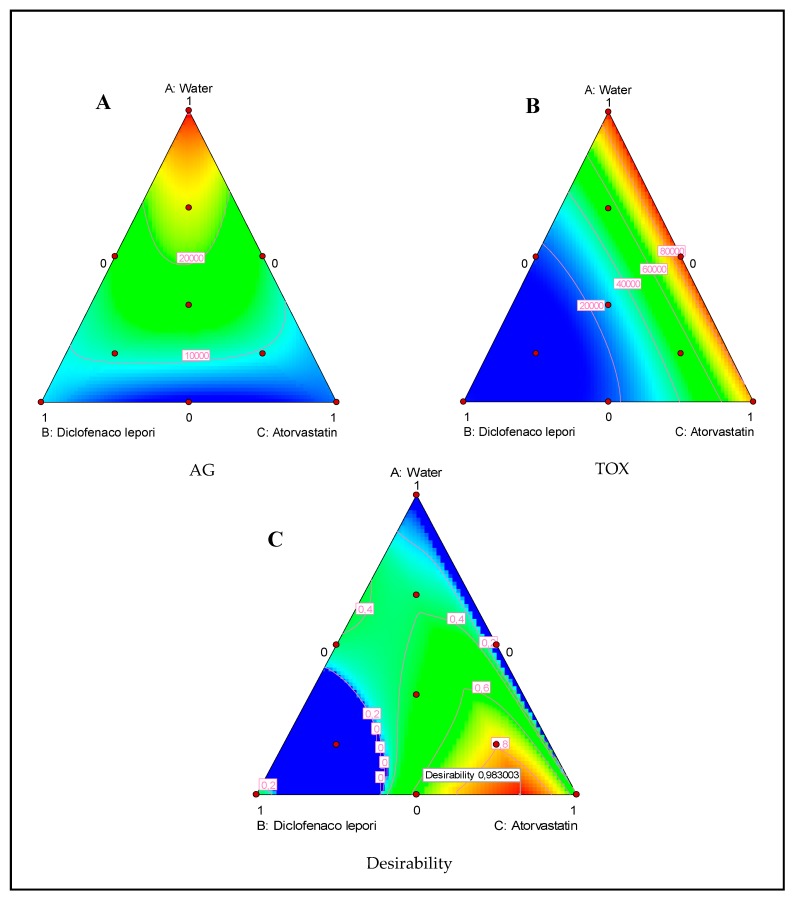
Ternary plots of mixture between water, Diclofenaco-lepori, and atorvastatin showing the effect of those components on the *A. castellanii* Neff growth (AG) (**A**) and induced toxicity (TOX) (**B**). Ternary plot for the desirability function showing the optimal drug mixture to inhibit the parasite without inducing toxic effects (**C**).

As for the second mixture plan composed of water, D-L, and atorvastatin, we could observe that both atorvastatin and the eye drop D-L presented a strong ability to inhibit the parasite growth, as shown in Figure 5A,B. Nevertheless, the D-L-based eye drop presented a high toxicity effect towards the tested murine macrophage cell lines.

### 2.3. Desirability Function

Candioti et al. (2014) reported that Derringer’s desirability function permits the analyst to obtain the experimental conditions (factor levels) to reach, simultaneously, the optimal value for all of the evaluated variables [18]. The desirability profile for optimum drug solution indicates that the maximum desirability of 1 (in a scale of 0–1) can be achieved with 3.1% water, 30.5% Optiben, and 66.4% atorvastatin for the Optiben mixture design and 17.6% Diclofenaco-lepori and 82.4% atorvastatin for the Diclofenaco-lepori mixture design. Under these conditions, we could increase the elimination of the parasite while causing low toxicity levels, as shown in Figure 4C and Figure 5C.

## 3. Materials and Methods

### 3.1. Chemicals

Optiben is a commercial eye drop prescribed in the case of dry eye syndrome. Its amoebicidal activity against *Acanthamoeba* spp. has already been reported in previous works [8]. Diclofenaco-lepori (1 mg/mL) is a commercial eye drop prescribed in the case of ocular inflammation. It contains diclofenac sodium (DC) as a principal active substance. DC is a nonsteroidal anti-inflammatory drug approved by the US Food and Drug Administration for ophthalmic use [19]. Atorvastatin is a hypolipidemic agent that is widely used to lower cholesterol levels, and it was purchased from Sigma-Aldrich Chemistry Ltd (Madrid, Spain). Atorvastatin was used at a concentration of 20 µM.

### 3.2. Experimental Design

The experimental mixture design was used to study the effect of mixing an eye drop and atorvastatin to inhibit *Acanthamoeba castellanii* Neff and murine macrophage growth. The 10 points or experiments consisted of three single component systems, three binary mixtures, and four ternary mixtures (Figure 1). The fluorescence for amoebicidal and cytotoxicity activities, which were selected as the responses for the combination of the independent variables, are presented in Table 1 and Table 3.

Once the experiments were performed, in order to correlate the response variables to the independent variables, a defined model was fitted. According to the analysis of variance, the regression coefficients of individual linear and interaction terms were determined. The regression coefficients were then used to make statistical calculations to generate ternary plots from the regression models. For each experiment, the total proportion was equal to 1. The maximum amount of each mixture component was fixed according to the data of a preliminary study (date not shown).

### 3.3. In Vitro Effect against the Trophozoite Stage of Acanthamoeba castellanii *Neff*

The amoebicidal activity of the different mixtures were evaluated against the American Type Culture Collection of *Acanthamoeba castellanii* Neff (ATCC 30010). The strain was axenically grown in PYG medium (0.75% (*w*/*v*) proteose peptone, 0.75% (*w*/*v*) yeast extract, and 1.5% (*w*/*v*) glucose) containing 40 μg gentamicin mL^−1^ (Biochrom AG, Cultek, Granollers, Barcelona, Spain).

The anti-*Acanthamoeba* activity of the different combinations were determined using the AlamarBlue^®^ assay [20]. Briefly, *Acanthamoeba* strain was seeded in duplicate on a 96-well microtiter plate with 50 μL from a stock solution of 5 × 10^4^ cells mL^−1^. Amoebae were allowed to adhere for 15 min, and their progress was checked using a Leika DMIL inverted microscope (Leika, Wetzlar, Germany). After that, 50 μL of each mixture were added to each well. Finally, the AlamarBlue Assay Reagent^®^ (Biosource, Europe, Nivelles, Belgium) was placed into each well at an amount equal to 10% of the medium volume. Test plates containing AlamarBlue were then incubated for 96 h at 28 °C with slight agitation, and the emitted fluorescence was examined with an Enspire microplate reader (PerkinElmer, Boston, MA, USA) at 570/585 nm.

### 3.4. Cytotoxicity Activity

The cytotoxicity of each mixture was evaluated after 24 h incubation of J774-A1 cell line murine macrophage, which has been previously established in our laboratory as a good marker of in vitro-induced toxicity at 37 ˚C in a 5% CO_2_ humidified incubator. The viability of the macrophages was determined with the AlamarBlue assay and the emitted fluorescence was examined with an Enspire microplate reader (PerkinElmer, Boston, Massachusetts, USA) at 570/585 nm, as previously described [21].

### 3.5. Statistical Analysis

Design-Expert 10 software (StatEase, MI, USA) was used to provide experimental designs, model building, the calculation of equations, and data analysis. Significant differences (*p* < 0.05, Duncan’s multiple-range test) between means were determined using GraphPad Prism 8 software (San Diego, CA, USA).

## 4. Conclusions

Statistical analysis showed that the obtained model adequately represented the experimental data, with a coefficient of multiple determination (*R*^2^) higher than 0.95. Moreover, the augmented simplex-centroid design was enough to describe and to predict the activity of the different drugs used in this study. Furthermore, the interaction between atorvastatin and each of the tested eye drops presented a negative coefficient against the trophozoite stage of *Acanthamoeba castellanii* Neff, hence, demonstrating the presence of synergy between both drugs. In conclusion, using the mixture design allowed us to enhance the anti-*Acanthamoeba* activity for both mixtures while lowering the induced toxicity caused by the Diclofenaco-lepori plan. Therefore, novel therapeutic combinations using experimental design (namely, the mixture design) seem to be feasible in order to reduce the number of experiments needed.

## Figures and Tables

**Table 1:** Coordinate setting for drug proportion based on the 10-point augmented simplex-centroid design and results from growth fluorescence for *Acanthamoeba* and macrophages (water, Optiben, and atorvastatin).

N° Exp	Drug Proportion			Fluorescence Growth (RFU)	
	Water	Optiben	Atorvastatin	AG	TOX
1	1	0	0	30,226	89,460
2	0	1	0	14,659	64,177
3	0	0	1	6349	80,663
4	0.5	0.5	0	25,487	88,579
5	0.5	0	0.5	13,385	83,238
6	0	0.5	0.5	5570	95,519
7	0.333333	0.333333	0.333333	14,548	93,556
8	0.666667	0.166667	0.166667	2,3785	8,5341
9	0.166667	0.666667	0.166667	1,6414	9,2424
10	0.166667	0.166667	0.666667	6489	9,4318

RFU: Relative Fluorescence Units. AG: *Acanthamoeba* Growth. TOX: Toxicity against murine macrophages.

**Table 2:** Regression coefficients and coefficients of determination (R2) obtained from mixture design of Atorvastatin, Optiben and water and their effect on amoebicidal activity (AA) and Macrophage cytotoxicity (MC).

Coefficients	Atorvastatin and Optiben	
Linear	AG (RFU)	TOX (RFU)
b_1_	30,443.9 ***	87,655.7 ^NS^
b_2_	14,982.5 ***	65,486 ^NS^
b_3_	5820.97 ***	81,475.4 ^NS^
Interaction		
b_12_	−14,721.1 **	46,051.3 ^NS^
b_13_	−18766 **	−9277.93 ^NS^
b_23_	−18,662.3 **	96,638.7 **
b_123_	19,076.3 ^NS^	39,874.8 ^NS^
R^2^	0.9938	0.9860

Subscripts: 1 = Water; 2 = Optiben; 3 = Atorvastatin, Significance levels: *** = *p* < 0.001; ** = *p* < 0.01; NS: non significative; AG: Acanthamoeba growth; TOX: Induced toxicity.

**Table 3:** Coordinate setting for drug proportion based on the 10-point augmented simplex-centroid design and results from growth fluorescence for *Acanthamoeba* and induced toxicity (water, D-L, and atorvastatin).

N° Exp	Drug Proportion			Fluorescence Growth (RFU)	
	Water	D-L	Atorvastatin	AG	TOX
1	1	0	0	30,226	89,460
2	0	1	0	8736	11,078
3	0	0	1	6349	80,663
4	0.5	0.5	0	15,555	14,597
5	0.5	0	0.5	13,385	90,265
6	0	0.5	0.5	1862	16,942
7	0.333333	0.333333	0.333333	18,491	28,815
8	0.666667	0.166667	0.166667	23,000	52,002.5
9	0.166667	0.666667	0.166667	8989	12,222.5
10	0.166667	0.166667	0.666667	8530	44,190

RFU: Relative Fluorescence Units. AG: *Acanthamoeba* Growth. TOX: Toxicity against murine macrophages.

**Table 4:** Regression coefficients (*R*^2^) and coefficients of determination obtained from mixture design of atorvastatin, Diclofenaco-lepori, and water and their effect on amoebicidal activity (AA) and induced cytotoxicity (IC).

	Atorvastatin and Diclofenaco-Lepori	
Linear	AG (RFU)	TOX (RFU)
b_1_	30,502.8 ***	88,829 ***
b_2_	8192.76 ***	13,389.3 ***
b_3_	5185.76 ***	78,487.8 ***
Interaction		
b_12_	−16,236.9 ^NS^	−139,328 **
b_13_	−17,382.9 **	15,201.7 ^NS^
b_23_	−22,134.9 **	−115,442 **
b_123_	236,784	−169,735 ^NS^
R^2^	0.9882	0.9874

Subscripts: 1 = Water; 2 = Diclofenaco-lepori; 3 = atorvastatin. ^b^ Significance levels: *** = *p* < 0.001; ** = *p* < 0.01; ^NS^: non-significant. AG: *Acanthamoeba* growth. TOX: Induced toxicity.

## References

[1] Vijayakumar R. (2018). Isolation, identification of pathogenic Acanthamoeba from drinking and recreational water sources in Saudi Arabia. J. Adv. Veter. Anim. Res..

[2] Reyes-Batlle M., Zamora-Herrera J., Vargas-Mesa A., Valerón-Tejera M.A., Wagner C., Martín-Navarro C.M., López-Arencibia A., Sifaoui I., Martínez-Carretero E., Valladares B. (2016). Acanthamoeba genotypes T2, T4, and T11 in soil sources from El Hierro island, Canary Islands, Spain. Parasitol. Res..

[3] Reyes-Batlle M., Todd C.D., Martín-Navarro C.M., López-Arencibia A., Cabello-Vílchez A.M., González A.C., Córdoba-Lanús E., Lindo J.F., Valladares B., Piñero J.E. (2014). Isolation and characterization of Acanthamoeba strains from soil samples in Gran Canaria, Canary Islands, Spain. Parasitol. Res..

[4] Motavalli M., Khodadadi I., Fallah M., Maghsood A.H. (2018). Effect of oxidative stress on vital indicators of *Acanthamoeba castellanii* (T4 genotype). Parasitol. Res..

[5] Castro-Artavia E., Retana-Moreira L., Lorenzo-Morales J., Abrahams-Sandí E. (2017). Potentially pathogenic *Acanthamoeba* genotype T4 isolated from dental units and emergency combination showers. Memórias Do Inst. Oswaldo Cruz.

[6] Grün A.-L., Stemplewitz B., Scheid P.L. (2014). First report of an *Acanthamoeba* genotype T13 isolate as etiological agent of a keratitis in humans. Parasitol. Res..

[7] Piñero J.E., Sifaoui I., Martín-Navarro C.M., López-Arencibia A., Reyes-Batlle M., Valladares B., Maciver S.K., Lorenzo-Morales J. (2019). Optimized combinations of statins and azoles against *Acanthamoeba trophozoites* and cysts in vitro. Asian Pac. J. Trop. Med..

[8] Sifaoui I., Reyes-Batlle M., López-Arencibia A., Chiboub O., Rodríguez-Martín J., Rocha-Cabrera P., Valladares B., Piñero J.E., Lorenzo-Morales J. (2018). Toxic effects of selected proprietary dry eye drops on *Acanthamoeba*. Sci. Rep..

[9] Lewis G.A., Mathieu D., Phan-Tan-Luu R. (1998). Pharmaceutical Experimental Design.

[10] Mura P., Gratteri P., Faucci M.T. (2002). Compatibility Studies of Multicomponent Tablet Formulations. DSC and experimental mixture design. J. Therm. Anal. Calorim..

[11] Scheffé H. (1958). Experiments with Mixtures. J. R. Stat. Soc..

[12] Dash S., Kumar A., Mandal B.N., Lal K., Kumar D. (2018). Experiments with mixtures. Bhartiya Krishi Anusandhan Patrika.

[13] Madgulkar A., Kadam S., Pokharkar V. (2009). Development of Buccal Adhesive Tablet with Prolonged *Antifungal* activity: Optimization and ex vivo Deposition Studies. Indian J. Pharm. Sci..

[14] Saoudi S., Chammem N., Sifaoui I., Jiménez I.A., Morales J.L., Piñero J.E., Bouassida-Beji M., Hamdi M., Bazzocchi I.L. (2017). Combined effect of carnosol, rosmarinic acid and thymol on the oxidative stability of soybean oil using a simplex centroid mixture design. J. Sci. Food Agric..

[15] Morales J.L., Martín-Navarro C.M., López-Arencibia A., Santana-Morales M.A., Afonso-Lehmann R.N., Maciver S.K., Valladares B., Martínez-Carretero E. (2010). Therapeutic Potential of a Combination of Two Gene-Specific Small Interfering RNAs against Clinical Strains of Acanthamoeba▿. Antimicrob. Agents Chemother..

[16] Rampersad S.N. (2012). Multiple Applications of Alamar Blue as an Indicator of Metabolic Function and Cellular Health in Cell Viability Bioassays. Sensors.

[17] Ayaki M., Yaguchi S., Iwasawa A., Koide R. (2008). Cytotoxicity of ophthalmic solutions with and without preservatives to human corneal endothelial cells, epithelial cells and conjunctival epithelial cells. Clin. Exp. Ophthalmol..

[18] Candioti L.V., De Zan M.M., Cámara M.S., Goicoechea H.C. (2014). Experimental design and multiple response optimization. Using the desirability function in analytical methods development. Talanta.

[19] Asasutjarit R., Theerachayanan T., Kewsuwan P., Veeranondha S., Fuongfuchat A., Ritthidej G.C. (2017). Gamma sterilization of diclofenac sodium loaded- N-trimethyl chitosan nanoparticles for ophthalmic use. Carbohydr. Polym..

[20] Martín-Navarro C.M., Morales J.L., Cabrera-Serra M.G., Rancel F., Coronado-Álvarez N.M., Piñero J.E., Valladares B. (2008). The potential pathogenicity of chlorhexidine-sensitive *Acanthamoeba* strains isolated from contact lens cases from asymptomatic individuals in Tenerife, Canary Islands, Spain. J. Med. Microbiol..

[21] Sifaoui I., López-Arencibia A., Martín-Navarro C.M., Reyes-Batlle M., Mejri M., Valladares B., Lorenzo-Morales J., Abderabba M., Piñero J.E. (2017). Selective activity of Oleanolic and Maslinic Acids on the Amastigote form of *Leishmania* Spp.. Iran. J. Pharm. Res. IJPR.

